# Impact of sample preparation upon intracellular metabolite measurements in 3D cell culture systems

**DOI:** 10.1007/s11306-019-1551-0

**Published:** 2019-06-12

**Authors:** Caroline Mathon, David Bovard, Quentin Dutertre, Sandra Sendyk, Mark Bentley, Julia Hoeng, Arno Knorr

**Affiliations:** 1 PMI R&D, Philip Morris Products S.A., Quai Jeanrenaud 5, 2000 Neuchâtel, Switzerland; 2 Present Address: Unit of Toxicology, CURML, Lausanne–Geneva, Switzerland

**Keywords:** Cell culture, Effectiveness, Extraction, Intracellular metabolites, LC-HRMS, Sample preparation

## Abstract

**Introduction:**

Interest in cell culture metabolomics has increased greatly in recent years because of its many potential applications and advantages (e.g., in toxicology). The first critical step for exploring the cellular metabolome is sample preparation. For metabolomics studies, an ideal sample preparation would extract a maximum number of metabolites and would enable reproducible, accurate analysis of a large number of samples and replicates. In addition, it would provide consistent results across several studies over a relatively long time frame.

**Objectives:**

This study was conducted to evaluate the impact of sample preparation strategies on monitoring intracellular metabolite responses, highlighting the potential critical step(s) in order to finally improve the quality of metabolomics studies.

**Methods:**

The sample preparation strategies were evaluated by calculating the sample preparation effect, matrix factor, and process efficiency (PE) for 16 tobacco exposition-related metabolites, including nicotine, nicotine-derived nitrosamine ketone, their major metabolites, and glutathione, using isotopically-labelled internal standards. Samples were analyzed by liquid chromatography (LC) coupled to high-resolution mass spectrometry (HRMS).

**Results:**

A sample drying step increased losses or variability for some selected metabolites. By avoiding evaporation, good sample preparation recovery was obtained for these compounds. For some metabolites, the cell or culture type impacted PE and matrix factor.

**Conclusion:**

In our sample preparation protocol, the drying–reconstitution step was identified as the main cause of metabolite losses or increased data variability during metabolomics analysis by LC-HRMS. Furthermore, PE was affected by the type of matrix. Isotopologue internal standards fully compensate losses or enhancements.

## Introduction

Characterization of the cellular metabolome, represented by intermediates and end products of all enzymatic reactions in a cell, enables the evaluation of cellular biochemical activity/networks. This information can predict molecular physiology and phenotypic changes in an organism (Töpfer et al. 2015). Stimuli or changes (e.g., environment, virus, food intake, smoking) induce responses at the metabolite level because of upstream modifications to DNA, mRNA, and proteins (Ivanisevic et al. 2013; Vinaixa et al. 2016; Worley and Powers 2013).

Metabolomics approaches may be categorized as follows: (i) An untargeted approach encompasses as many metabolites as possible to provide a comprehensive analysis. For these global analyses, the entire workflow must be non-selective, reproducible, and unbiased in order to preserve the integrity of cellular metabolites and include as many metabolites as possible (Cala and Meesters 2017; Ulmer et al. 2015). Metabolite abundances or relative responses are reported. (ii) A targeted approach is focused on a limited list of metabolites. In this case, sample preparation and analytical methods are optimized for specific compounds, which are absolutely quantified using calibration curves and internal standards for each metabolite. (iii) A semi-targeted approach lies between the targeted and untargeted approaches. Many metabolites are screened and semi-quantified, using single calibration curves for multiple compounds (Broadhurst et al. 2018).

**Fig. 1:**
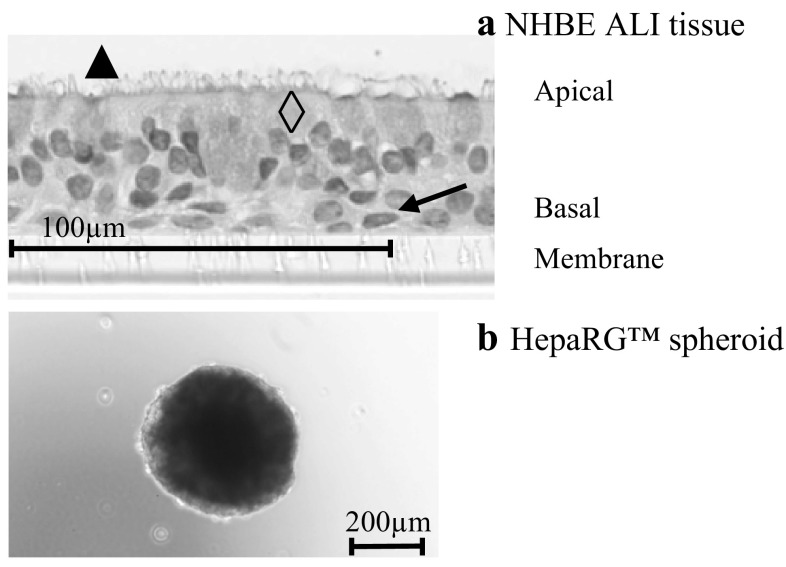
**a** Representative histological section of NHBE ALI tissues, stained with hematoxylin, eosin, and Alcian blue. Magnification ×20. The in vitro tissue was composed of ciliated cells (black triangle), goblet cells (empty diamond), and basal cells (black arrow). **b** Representative bright-field images of HepaRG™ spheroid. Magnification ×10

For toxicological assessment, changes in metabolic profiles and their relationship to various biochemical pathways are investigated. Untargeted approaches have been used for this purpose, for instance, to explore the global impact of environmental chemical contaminants on earthworms (Griffith et al. 2018) or on human cells (Hartung et al. 2017), or to assess the impact of tobacco exposure on in vitro systems (Iskandar et al. 2017; Kogel et al. 2016; Zanetti et al. 2017). For in vitro toxicology studies, two-dimensional (2D) cell cultures have been the most commonly investigated systems for decades. Currently, however, human three-dimensional (3D) tissue cultures are widely regarded as more relevant than 2D monolayer cell cultures for in vitro toxicological assessments. In 2D culture systems, the extracellular matrix and cell–cell and cell–matrix interactions, all essential for differentiation, proliferation, and cellular functions, are not present. In contrast, 3D cultures are sometimes composed of several cell types where cell–cell and cell–matrix interactions are formed, recreating physiological tissue structure, enabling cell differentiation, and finally, potentially increasing drug susceptibility (Bovard et al. 2017; Ravi et al. 2015). Several 3D tissues mimicking human organs are commercially available or can be generated by investigators in-house. Bronchial 3D organotypic tissues can be prepared starting from primary airway epithelial human cells grown at the air–liquid interface (ALI) and seeded on a collagen-based support: in these conditions, cells differentiate to form a functional bronchial tissue composed of basal, goblet, and ciliated cells (Fig. 1a) (Karp et al. 2002). Such 3D bronchial tissue models have morphological and metabolic characteristics that are comparable with those of large human airways (Baxter et al. 2015; Pezzulo et al. 2011). Furthermore, when bronchial tissue cultures are developed and maintained at the ALI, they can be exposed to various aerosols, including cigarette smoke or a candidate modified risk tobacco product aerosol, mimicking in vivo inhalation exposure (Iskandar et al. 2017). These toxicological investigations have greatly improved over the last few years thanks to the development of new 3D models that better mimic lung function. In addition to bronchial tissues, liver spheroids were also investigated, as the liver is the most metabolically active organ (Fathi et al. 2017). Liver function can be mimicked by liver spheroids composed of HepaRG™ cells (Fig. 1b). HepaRG™ cells are terminally differentiated hepatic cells derived from a human hepatic progenitor cell line, retaining many characteristics of primary human hepatocytes. The spheroid organization enables close contact between cells as well as polarization and formation of bile canaliculi (Alepee et al. 2014). In addition to these morphological advantages, 3D organization of liver cells leads to increased albumin secretion, the ability for cytochrome P450 family induction and longer cell survival than 2D cultures (Takahashi et al. 2015). As described by Groell et al. 3D cell cultures represent a perfect compromise between 2D and animal models (Groell et al. 2018).

For untargeted cellular metabolome characterization, sample preparation must be non-discriminative and precise. The protocol must also maintain sample stability, because intracellular metabolites can be rapidly degraded or metabolized by enzymatic reactions, and must minimize contaminants in both sample and detector (Drouin et al. 2018; Pinu et al. 2017). Precise results from single-time-point analyses or kinetic studies are necessary in order to obtain an accurate snapshot of the metabolome. Cell extracts must be rapidly and reproducibly quenched to stop metabolic activity to ensure consistent results across many samples and several studies over an extended time frame. Compound loss or modification caused by chemical or enzymatic reactions occurring during sample harvest, preparation, and analysis must be minimized. Changes occurring in the autosampler during chromatographic analysis can be monitored by comparing the signal responses with injection order and can be corrected using quality control (QC) samples or by including appropriate stable isotopically labelled internal standards (ISTD) (Broadhurst et al. 2018). ISTDs can be used to compensate for changes occurring at any time after they are added to the samples, even during sample preparation (Pinu et al. 2017). It is of paramount importance to minimize potential changes before the addition of ISTDs, which are difficult to evaluate, by decreasing duration of sampling and by placing samples into low temperature storage as soon as possible (Pinu et al. 2017).

To our knowledge, there are no harmonized procedures for sample preparation of 3D cell cultures for untargeted metabolomics. More generally, standard protocols for sample preparation have not been fully established, mostly because metabolomics is a rapidly evolving technique utilizing a broad range of analytical techniques and sample matrices and measuring metabolites with a wide variety of physical and chemical properties (Bi et al. 2013; Daskalaki et al. 2018; Ulmer et al. 2015). Nevertheless, one recent guideline for untargeted metabolomics analysis is available (Broadhurst et al. 2018). Although sample preparation is a crucial step, as it can affect the entire study outcome, it is often neglected, and evaluation of its impact is rarely considered (Cuykx et al. 2017; Dettmer et al. 2011; Martinovic et al. 2018; Römisch-Margl et al. 2012; Sapcariu et al. 2014). Intracellular metabolites were extracted using freeze-thawing cycles or homogenization, with and without a drying–reconstitution step. Sample extracts were analyzed by liquid chromatography (LC) coupled to high-resolution mass spectrometry (HRMS). To evaluate the reproducibility of the generic analytical approach, we chose 16 chemicals and eight ISTDs specific to our research area. These compounds (Table 1), all related to tobacco exposure, included nicotine, nicotine-derived nitrosamine ketone (NNK), and their major metabolites as well as glutathione (GSH), a biomarker of oxidative stress. Of these, nicotine, NNK, and their metabolites are either abundant in tobacco-derived aerosols (nicotine) or have carcinogenic potential (NNK) (Konstantinou et al. 2018). Understanding the metabolism pathways for xenobiotics, such as nicotine and its metabolites, the major markers for cigarette smoke exposure, requires precise metabolite analyses, either at individual time points or in kinetic studies. Based on this compound pallet, method was evaluated by calculating the effect of the sample preparation, matrix factor, and process efficiency (PE) for the selected metabolites and ISTDs for bronchial and hepatic 3D cell cultures.

**Table 1:** List of the major nicotine, NNK, and GSH metabolites investigated, with their corresponding abbreviations, ISTDs, CAS number, formula, molecular ion *m/z* in positive electrospray ionization, retention times, and suppliers

Categories	Metabolites	Abbreviations	ISTDs	CAS	Formula	*m/z* [M+H]^+^	t_R_	Suppliers
Nicotine	Cotinine D_3_	Cot D_3_	N/A	110952-70-0	C_10_H_5_D_7_N_2_O	180.12107	8.0	Sigma-Aldrich
	3-hydroxy-cotinine-*O*-β-glucuronide	OH Cot-*O*-gluc.	Cot D_3_	132929-88-5	C_16_H_20_N_2_O_8_	369.12924	3.9	TRC
	Cotinine	Cot	Cot D_3_	486-56-6	C_10_H_12_N_2_O	177.10224	8.0	Sigma-Aldrich
	Cotinine-*N*-oxide	Cot-*N*-Ox	Cot D_3_	36508-80-2	C_10_H_12_N_2_O_2_	193.09715	7.5	TLC PharmaChem
	Nicotine D_3_	Nic D_3_	N/A	69980-24-1	C_10_D_3_H_11_N_2_	166.14181	8.4	Sigma-Aldrich
	Nicotine	Nic	Nic D_3_	54-11-5	C_10_H_14_N_2_	163.12298	8.4	Sigma-Aldrich
	Nicotine-*N*-β-glucuronide D_3_	Nic-*N*-gluc. D_3_	N/A	329002-74-6	C_16_H_19_D_3_N_2_O_6_	342.17389	1.8	SCBT
	Nicotine-*N*-glucuronide	Nic-*N*-gluc.	Nic-*N*-Gluc. D_3_	152306-59-7	C_16_H_22_N_2_O_6_	339.15506	1.8	TRC
	Nicotine-*N*-oxide D_3_	Nic-*N*-ox D_3_	N/A	491-26-9	C_10_H_11_D_3_N_2_O	182.13672	8.8	TLC PharmaChem
	Nicotine-*N*-oxide	Nic-*N*-ox	Nic-*N*-ox D_3_	491-26-9	C_10_H_14_N_2_O	179.11789	8.8	TLC PharmaChem
	Nornicotine D_4_	Nornic D_4_	N/A	66148-18-3	C_9_H_8_ D_4_N_2_	153.13243	6.0	TRC
	Nornicotine	Nornic	Nornic D_4_	5746-86-1	C_9_H_12_N_2_	149.10732	6.0	Sigma-Aldrich
NNK	4-(Methylnitrosamino)-1-(3-pyridyl)-1-butanol D_3_	NNAL D_3_	N/A	1020719-61-2	C_10_H_12_D_3_N_3_O_2_	213.14253	8.4	TRC
	4-(Methylnitrosamino)-1-(3-pyridyl)-1-butanol	NNAL	NNAL D_3_	76014-81-8	C_10_H_15_N_3_O_2_	210.12370	8.4	TRC
	4-(Methylnitrosamino)-1-(3-pyridyl)-1-butanone	NNK	NNAL D_3_	64091-91-4	C_10_H_13_N_3_O_2_	208.10805	11.7	Sigma-Aldrich
	4-(Methylnitrosamino)-1-(3-pyridyl-*N*-oxide)-1-butanol	NNAL-*N*-ox	NNAL D_3_	85352-99-4	C_10_H_15_N_3_O_3_	226.11862	8.2	TRC
	4-(Methylnitrosamino)-1-(3-pyridyl-*N*-oxide)-1-butanone	NNK-*N*-ox	NNAL D_3_	76014-82-9	C_10_H_13_N_3_O_3_	224.10297	9.3	TRC
	4-Hydroxy-1-(3-pyridyl)-1-butanone D_2_	HPB D_2_	N/A	154603-21-1	C_9_H_9_D_2_NO_2_	168.09881	8.9	TRC
	1-(3-Pyridyl)-1,4-butane diol	Diol	HPB D_2_	76014-83-0	C_9_H_13_NO_2_	168.10191	3.2	TRC
	1-(3-Pyridyl)-1-butanol-4-carboxylic acid	Hydroxy acid	HPB D_2_	15569-97-8	C_9_H_11_NO_3_	182.08117	3.4	TRC
	1-(3-Pyridyl)-1-butanone-4-carboxylic acid	OPBA	HPB D_2_	4192-31-8	C_9_H_9_NO_3_	180.06552	9.0	TRC
	4-Hydroxy-1-(3-pyridyl)-1-butanone	HPB	HPB D_2_	59578-62-0	C_9_H_11_NO_2_	166.08626	8.9	TRC
GSH	Glutathione reduced ^13^C_2_^15^N	GSH ^13^C_2_^15^N	N/A	815610-65-2	^13^C_2_^15^N C_8_H_17_N_2_O_6_S	311.09483	2.7	Sigma-Aldrich
	Glutathione reduced	GSH	GSH ^13^C_2_^15^N	70-18-8	C_10_H_17_N_3_O_6_S	308.09108	2.7	Sigma-Aldrich

*ISTDs* isotopically labelled internal standards, *SCBT* Santa Cruz Biotechnology, *TRC* Toronto Research Chemicals

## Materials and methods

### Reagents and chemicals

LC coupled to mass spectrometry (MS)-grade water and methanol (LC/MS Chromasolv) were supplied by Honeywell Riedel-de Haën™ (Seelze, Germany). Analytical standards, ISTDs, and formic acid were supplied by Sigma Aldrich (St. Louis, MO, USA), Toronto Research Chemicals (Ontario, Canada), TLC PharmaChem (Ontario, Canada), or Santa Cruz Biotechnology (Dallas, TX, USA), depending on availability (details for analytes and ISTDs are presented in Table 1).

The standard and ISTD solutions (commercially obtained or prepared in the laboratory from powder), at approximately 1 mg/mL, were stored at − 20 °C and diluted in methanol/water (4:1 v/v) on the day of analysis for spiking mixtures and calibration curves. Calibration levels ranged over three orders of magnitude for each metabolite, and concentrations were selected based on MS sensitivity of each metabolite and its expected concentrations in the samples.

### HepaRG™ spheroids

HepaRG™ cells (Ref. HPRGC10, ThermoFisher Scientific) were first thawed in William’s E medium (Ref. 12551032, ThermoFisher) supplemented with GlutaMAX™ (Ref. 35050061, ThermoFisher) and HepaRG™ Thaw, Plate & General Purpose Medium Supplement (Ref. HPRG770, ThermoFisher). After thawing, 25,000 cells were seeded in each well of an ultra-low adhesion 96-well plate (Ref. 4520, Corning, NY, USA). The 96-well plates were kept in the incubator at 37 °C for 4 days before medium replacement. On the fourth day, cells formed dense aggregates with a visible extracellular matrix confirming spheroid formation. Thereafter, the cell culture medium was renewed every 2–3 days. Spheroids were used once mature, approximately 1 week after thawing HepaRG™ cells.

### NHBE ALI tissues

Normal human bronchial epithelial (NHBE) ALI tissues were prepared following a procedure described by STEMCELL Technologies (PneumaCult™ Medium, Document #29252; STEMCELL Technologies, Vancouver, Canada). Briefly, NHBE cells (Lonza, Basel, Switzerland) were cultured in T75-flasks using PneumaCult™-EX PLUS medium (Ref. 05040, STEMCELL Technologies) at 37 °C with 5% CO_2_ and 90% relative humidity. Once the cells were 80% confluent, they were detached from the flask using trypsin-ethylenediaminetetraacetic acid (EDTA) (Ref. CC-5034, Lonza), and 50,000 cells were seeded on a Collagen I-coated Transwell® insert (Corning®, Corning, NY, USA). Both apical and basal sides of the inserts were filled with PneumaCult™-EX PLUS medium and maintained for 3 days. Subsequently, the culture was airlifted by removing the apical medium; the basal medium was replaced with the PneumaCult™-ALI medium (STEMCELL Technologies). Tissues were used for experiments starting from day 28 after airlift.

### Sample preparation

**Fig. 2:**
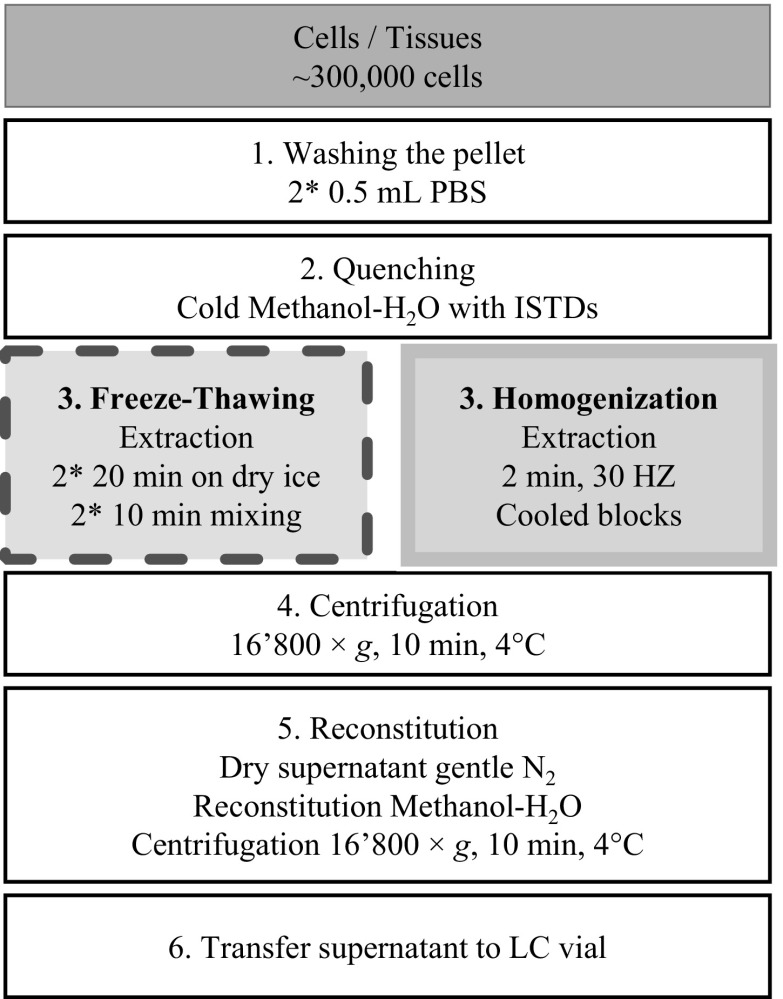
Sample preparation workflow for freeze-thawing- and homogenization-based procedures. In both protocols, all steps were common, other than the extraction step (step 3). Because of the significant signal decrease or variability caused by drying–reconstitution, this step (step 5) was skipped in subsequent experiments

The sample preparation workflow is shown in Fig. 2. Two extraction procedures were evaluated in parallel, based on either freeze-thawing or homogenization. Other than the extractions, all other steps were common to all samples.

After removal of the culture medium, cells were washed twice with 0.5 mL phosphate-buffered saline (PBS) without calcium chloride and magnesium chloride (Ref. D8537, Sigma).

For both extraction procedures, washed cell cultures were quenched with 150 µL cold (− 20 °C) methanol/water (4:1 v/v) containing the ISTDs and, for spiked samples, the standards. For the HepaRG™ spheroids, which were non-adherent, spheroids and solvent were transferred directly into an Eppendorf tube for further extraction. As the NHBE ALI tissues were adherent and not homogenous, tissues were harvested from the inserts after quenching by scraping into cold methanol/water, on ice.

#### Freeze-thawing extraction procedure

After quenching, cells were frozen using dry ice for 20 min and then agitated for 10 min at 30 °C and 800 rpm on a ThermoMixer C (Eppendorf, Schönenbuch, Switzerland). This process was repeated for two cycles.

#### Homogenization extraction procedure

Zirconium beads (1.4 mm) were added to the quenched cells, in Eppendorf tubes, and tubes were placed in cold homogenizer blocks (pre-cooled to −20 °C). Samples were disrupted with a tissuelyser (QIAGEN, Hombrechtikon, Switzerland) for 2 min at 30 Hz.

After extraction, to remove proteins and other cell debris, the extracts were centrifuged for 10 min at 16’800×*g* (4° C). Supernatants were transferred into vials (60 µL) for LC-HRMS analysis.

For the drying–reconstitution step, instead of transferring the aliquot extracts to LC-HRMS vials directly, aliquots (100 µL) were dried either under a gentle N_2_ stream at room temperature, or with a Speedvac at 30 °C (Christ, Osterode, Germany). The dried extracts were then reconstituted in 100 µL methanol/water, (4:1 v/v). After centrifugation for 10 min at 4 °C, 16’800×*g*, supernatants were transferred into vials (60 µL) for LC-HRMS analysis.

### Experimental design

**Fig. 3:**
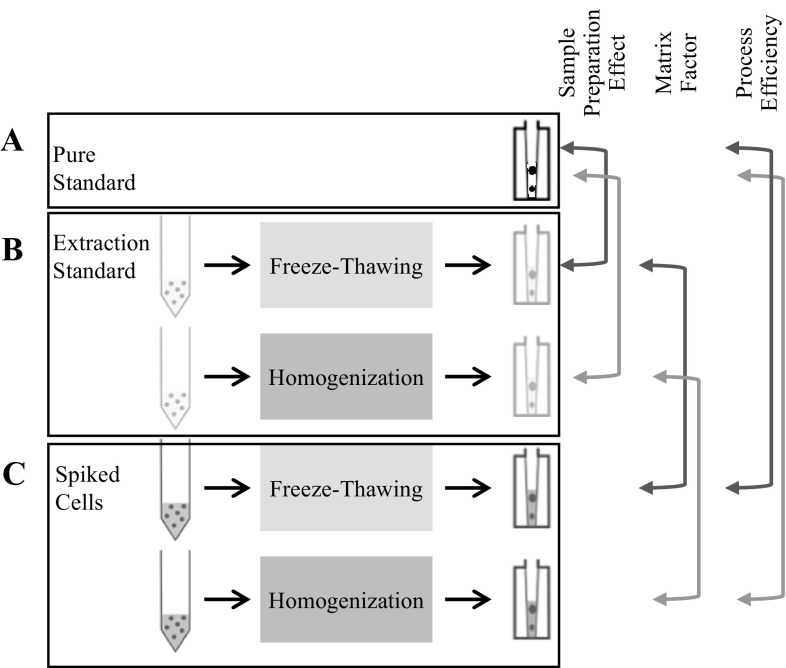
Assay procedure to evaluate sample preparation methods by measuring sample preparation effect (b/a), matrix factor (c/b), and PE (c/a). Adapted from Mathon et al. (2013) and Noga et al. (2018)

To evaluate sample preparation, two extraction protocols (i.e. freeze-thawing or homogenization), were performed and compared for standard solutions and for each cell type (i.e. NHBE ALI tissues and HepaRG™ spheroids) (Figs. 2, 3). For both protocols the extraction solvent was a mixture of methanol–water (80:20 v/v).

Concentrations measured in pure standard solutions (Fig. 3a) were compared to concentrations measured in standard solution going through the extractions (Fig. 3b), as well as concentrations measured in the spiked cells (Fig. 3c).

For each sub-group (*e.g., pure standard, spiked HepaRG cells extracted with freeze*–*thaw procedure*, *…*), six replicates were prepared and injected once.

### LC-HRMS instrumentation

All analyses were performed using an Accela 1250 ultra-high performance LC system coupled to a Q-Exactive HRMS equipped with a heated electrospray ionization source (Thermo Fisher Scientific, Bremen, Germany).

Metabolite separation was performed using two complementary stationary phases in series, comprising a Biobasic anion-exchange column (50 mm × 2.1 mm, 5 µm particle size, Thermo Scientific) followed by a Kinetex® pentafluorophenyl column (150 mm × 2.1 mm, 2.6 µm particle size, 100 Å, Phenomenex, Basel, Switzerland). Samples were analyzed in positive ionization mode using 10 mM ammonium formate buffer, pH 3.5 (eluent A), and methanol/water/formic acid (95:5:0.1, v/v/v) (eluent B). The elution gradient was: held at 99% A for 2 min, 99% A to 55% A in 8 min, then decreasing to 0% A in 3 min and held at 100% B for 2 min. Initial conditions were restored between samples by returning to 99% A over 1 min, followed by equilibration for an additional 4 min. The flow rate was 0.28 mL/min, the LC column oven was maintained at 40 °C, the injection volume was 1 µL and the autosampler was maintained at 10 °C.

The HRMS system was calibrated and tuned according to the manufacturer’s recommendations using the tune mix (REF 88323, ESI positive ion calibration solution, Fisher Scientific, Loughborough, UK) Nitrogen (purity > 99.999%) was used as a sheath gas and auxiliary gas at a flow of 50 and 15 arbitrary units, respectively. Auxiliary gas and capillary temperatures were 350 °C and 300 °C, respectively. Spray voltage was 3.00 kV.

Full scan acquisition mode (*m/z* 60–900) at a mass resolving power of 70,000 (full width at half maximum, FWHM) at *m/z* 200 with an automatic gain control (AGC) target set at 5e^5^, and a maximum inject time of 150 ms was used. For tandem mass spectrometry (MS/MS) metabolite identification, the following conditions were used: data-dependent MS/MS top three of each scan at 17,500 (FWHM) at *m/z* 200, with an AGC target set at 5e^4^ and a maximum injection time of 150 ms, with stepped normalized collision energies set at 25, 50, and 75 eV with an isolation window of 1 *m/z*.

### Data processing and data analysis

Metabolites detection, integration and quantification were done with TraceFinder 3.0 software (Thermo Fisher Scientific).

**Fig. 4:**
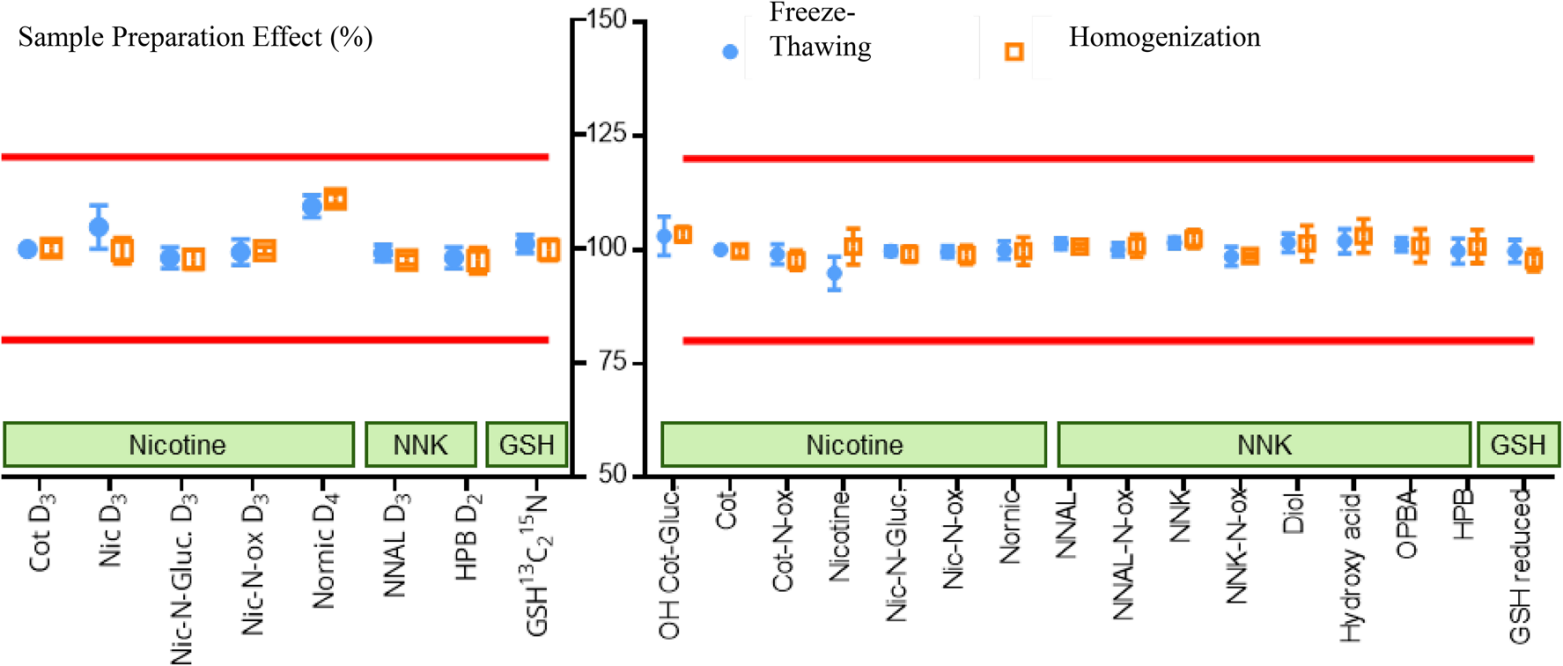
Sample preparation effect of ISTDs and analytes with no matrices for three classes of metabolites: nicotine, NNK, and GSH. The data are mean sample preparation effect values for freeze-thawing-based extraction (filled dots) and homogenization-based extraction (unfilled squares). Bars show standard deviations (n = 6). Analyte recovery values were calculated based on analyte concentrations, as quantified using ISTDs. *Cot* cotinine, *Diol* 1-(3-pyridyl)-1,4-butane diol, *Gluc*. glucuronide, *GSH* glutathione, *HPB* 4-hydroxy-1-(3-pyridyl)-1-butanone-4,4, *Hydroxy acid* 1-(3-pyridyl)-1-butanol-4-carboxylic acid, *Nic* nicotine, *NNAL* 4-(methylnitrosamino)-1-(3-pyridyl)-1-butanol, *NNK* 4-(methylnitrosamino)-1-(3-pyridyl)-1-butanone, *OPBA* 1-(3-pyridyl)-1-butanone-4-carboxylic acid, *Ox* oxide

**Fig. 5:**
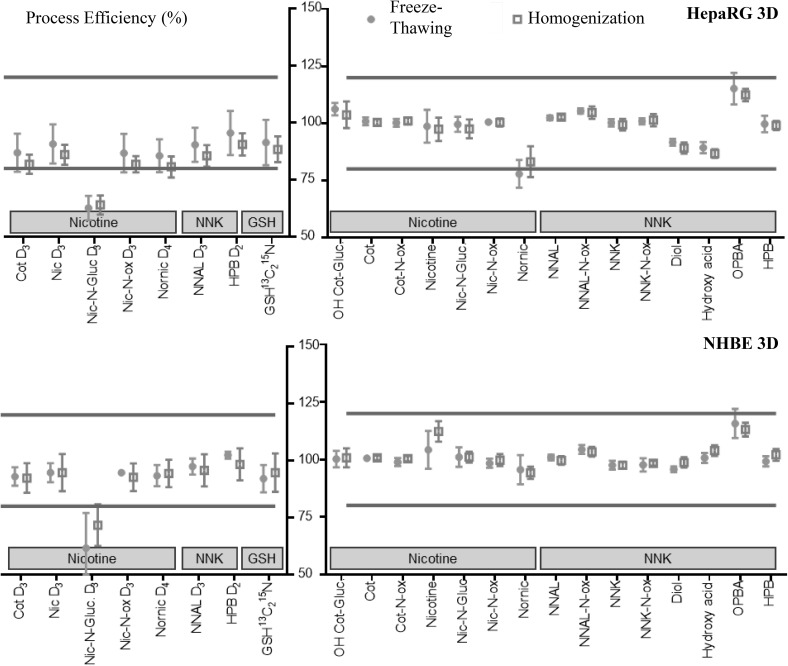
PE (C/A) values for ISTDs and analytes for the three classes of metabolites: nicotine, NNK, and GSH. Samples were obtained from 3D tissues, HepaRG™ spheroids (upper) or NHBE ALI tissues (lower). Data are means for freeze-thawing-based extraction (filled dots) and homogenization-based extraction (unfilled squares). Bars show standard deviations (n = 6). Data for analytes were based on concentrations quantified using ISTDs. *Cot* cotinine, *Diol* 1-(3-pyridyl)-1,4-butane diol, *Gluc*. glucuronide, *GSH* glutathione, *HPB* 4-hydroxy-1-(3-pyridyl)-1-butanone-4,4, *Hydroxy acid* 1-(3-pyridyl)-1-butanol-4-carboxylic acid, *Nic* nicotine, *NNAL* 4-(methylnitrosamino)-1-(3-pyridyl)-1-butanol, *NNK* 4-(methylnitrosamino)-1-(3-pyridyl)-1-butanone, *OPBA* 1-(3-pyridyl)-1-butanone-4-carboxylic acid, *Ox* oxide

Calculations, tests (Holm–Sidak test, α 0.05, heteroscedastic) and graphs (Figs. 4, 5) were performed using GraphPad Prism 7.0 software (GraphPad, CA, USA).

## Results and discussion

For untargeted metabolomics analysis, an ideal preparation method would extract the maximum number of metabolites from a large number of samples with high precision. Sample preparation protocols can be influenced by cell culture properties (adherent or suspended, 2D or 3D cultures) as well as cell types, the physico-chemical properties of the metabolites, and the techniques used for their detection. 3D cell cultures were used for this investigation because they are now routinely used for toxicological assessment, and a new extraction approach was required compared with 2D cell cultures.

Understanding the metabolism pathways for xenobiotics, such as nicotine and its metabolites, the major markers for cigarette smoke exposure, requires precise metabolite analyses, either at individual time points or in kinetic studies. To evaluate the precision of a non-discriminative analytical approach for intracellular metabolite analysis, sample preparation effect, matrix factor, and PE were calculated for 16 metabolites of interest (i.e., nicotine, NNK, and their major metabolites) and eight ISTDs related to our research scope. Some ISTDs were used for more than one metabolite, based on their chemical and physical properties (Table 1).

The addition of ISTDs, which were not present endogenously in the samples, enabled evaluation of any changes, including signal suppression or enhancement caused by sample preparation or analysis, in each extract (Broadhurst et al. 2018; Noga et al. 2018). As reported by Broadhurst et al. another advantage of including ISTDs in all samples was to enable real-time tracking of sample responses during data collection (mass accuracy and resolution, retention time, peak intensity, and peak shape). This is an advantage over the use of QC samples, which are not evaluated until the end of a batch or the entire study analysis (Broadhurst et al. 2018). Absolute quantification of compounds using ISTDs enables correction for procedural variations occurring at any time after the ISTDs were added to the sample. In our study, ISTDs were added at the beginning of sample preparation and during cell quenching and extraction to address any post-harvesting changes in target metabolites. Results are presented for analyte concentrations quantified using the ISTDs as well as for the ISTD responses themselves.

A rapid, simple sample preparation method, performed at a low temperature to minimize changes in metabolites, was evaluated using two different extraction methods.

### Assay procedure

To evaluate sample preparation and the analytical method, selected metabolites were injected as pure standards, as standards without matrix undergoing sample extraction procedures, and as standards with matrix undergoing sample extraction procedures (Fig. 3). One advantage of cell culture is that the environment is controlled, limiting sample-to-sample variations and enabling robust statistics with a limited number of replicates (n = 6 in this study) (Dudzik et al. 2018).

To evaluate sample preparation effects, results obtained with pure standard solutions, containing the 16 metabolites and the eight ISTDs, were compared with those for the same solution subjected to two sample preparation procedures, freeze–thawing and homogenization extraction procedures, without matrix (Fig. 3).

In an analogous manner, the impact of the matrix, calculated as a matrix factor, was determined by comparing the results obtained for extracted standard solutions with those of metabolites and ISTDs spiked on cells during the quenching step (Fig. 3).

Finally, PE was determined by comparing results from injected pure standard solutions with those from spiked cell samples undergoing the complete sample preparation process (Fig. 3).

For endogenous metabolites such as GSH, always present in the intracellular metabolome, PE was measured using the ISTD only.

### Cell washing, quenching, and extraction

To evaluate the intracellular metabolome, medium was removed, and cells were washed to remove medium components known to affect the cellular metabolome (Daskalaki et al. 2018). The washed 3D cell cultures were then quenched. During all processing, cells were cooled on either ice or dry ice.

For the adherent and not homogenous NHBE ALI tissues, cells were harvested from the inserts by scraping into cold methanol/water, on ice. Scraping limited metabolite leakage, as previously demonstrated with EDTA treatment (Dudzik et al. 2018). Samples were then directly extracted using two freeze-thaw cycles or one homogenization cycle, as described in Sect. 2.5.

### Drying and reconstitution

Drying and reconstitution is commonly used to concentrate samples in order to increase the number of metabolites detected. Additionally, samples can be reconstituted in solvents other than the extraction solution for compatibility with the analytical method.

The impact of the drying step used to concentrate the extracts on metabolite detection was quantified by reconstituting the samples in the same solvent (e.g., the extraction mixture [methanol/water, 4:1 v/v]) (Fig. 2). The concentration procedure substantially decreased the responses for nicotine, nornicotine, GSH, and their corresponding ISTDs. For instance, after overnight drying nornicotine D_4_ showed a very good relative standard deviation (RSD) in the calibration solutions (RSD = 3%), while for samples been through the extraction processes (freeze-thawing or homogenization) the RSD were high (between 24 and 72%), for both cell types. Additionally, the nicotine D_3_ PEs were low (between 18% for HepaRG™ spheroids after homogenization, to 54% for NHBE ALI tissues after freeze-thawing). While drying overnight led to consistent signal decreases in all samples, a duration of 2 h resulted in high variability in the data. For example, nicotine D_3_ was not detected in samples after overnight drying, while nicotine D_3_ was detected in 61% of the samples dried 2 h.

High response variability or absence of the ISTD drastically impacts quantification of the metabolites, if they are detected. Because of the significant signal decrease or variability caused by drying, this step was not included in subsequent experiments. After extraction and centrifugation, samples were instead directly transferred to LC-HRMS vials for analyses (Fig. 2).

If a drying step is required to concentrate an extract or modify solvent composition, ISTDs must be added to correct for any metabolite changes caused by drying.

### Method performance

To evaluate sample preparation effect without matrix, the ratio between compound responses observed in a standard solution processed by the two extraction procedures were compared with those in pure standard solutions (Fig. 3).

There were no significant differences between the two extraction procedures in analysis of the ISTDs (Holm–Sidak test, α 0.05, heteroscedastic). The mean sample preparation effect values (n = 6) for the ISTDs were between 97.3 ± 2.8% (for 4-hydroxy-1-(3-pyridyl)-1-butanone D_2_ [HPB-D_2_]) and 110.8 ± 1.2% (for nornicotine D_4_), both after the homogenization process, as shown in Fig. 4 (left). The analytes were quantified using their corresponding ISTDs (Table 1), and no significant differences were observed between the two extraction processes (Holm–Sidak test, α 0.05, heteroscedastic). The mean sample preparation effect values (n = 6) for the analytes were between 94.8 ± 3.6% for nornicotine (after the freeze-thawing process) and 103.3 ± 1.6% for 3-hydroxy-cotinine-*O*-glucuronide (OH Cot-Gluc, after the homogenization process), as shown in Fig. 4 (right).

As detailed above, without matrix, sample preparation recoveries were good for ISTDs and analytes, with accurate, reproducible results and no significant differences between the two extraction procedures.

To evaluate the impact of extraction procedures on metabolites from NHBE ALI tissues and HepaRG™ spheroids, PE was calculated by comparing results from spiked cell cultures to those from pure standard solutions (Fig. 3).

For the ISTDs, PE values were calculated by dividing the area obtained for each ISTD from spiked matrices by that from pure standard solutions. For HepaRG™ spheroids, PE values were between 62.7 ± 5.3% (nicotine-*N*-glucuronide D_3_, Nic-*N*-Gluc. D_3_) and 95.5 ± 9.9% (HPB D_2_) using homogenization (Fig. 5, upper left). For NHBE ALI tissues, Nic-*N*-Gluc. D_3_ was detected with lowest efficiencies (61.7 ± 15.3% and 71.7 ± 9.1% for homogenization and freeze-thawing, respectively), as shown in Fig. 5 (lower left).

For the analytes, PE values were calculated using concentrations quantified with ISTDs in spiked matrices divided by those measured in pure standard solutions. Of the metabolites, nornicotine had the lowest PE value in HepaRG™ spheroids (83.2 ± 6.7% and 77.8 ± 6.0% for homogenization and freeze-thawing, respectively) as well as in NHBE ALI tissues (94.2 ± 2.6% and 95.5 ± 6.3% for homogenization and freeze-thawing, respectively) (Fig. 5, right). The highest PE values were obtained in both matrices and extraction procedures for 1-(3-pyridyl)-1-butanone-4-carboxylic acid (OPBA) in NHBE ALI tissues (113.0 ± 3.0% and 115.7 ± 6.3% for homogenization and freeze-thawing, respectively) and in HepaRG™ spheroids (112.3 ± 2.7% and 115.2 ± 6.9% for homogenization and freeze-thawing, respectively) (Fig. 5, right).

The results for OPBA analysis were explained by the use of a non-isotopologue ISTD, HPB D_2_, a stable isotope-labelled compound with a molecular structure that is close, but not exactly the same. When the ISTDs and analytes were isotopologues, the analyte PE values were nearly 100%, as shown for nicotine-*N*-glucuronide in HepaRG™ spheroids (97.5 ± 4.1% and 99.5 ± 3.3% for homogenization and freeze-thawing, respectively) and in NHBE ALI tissues (101.0 ± 2.4% and 101.0 ± 4.2% for homogenization and freeze-thawing, respectively). In contrast, Nic-*N*-Gluc D_3_ analysis had the lowest PE values in homogenized HepaRG™ spheroids (62.7 ± 5.3%) and in NHBE ALI tissues (61.7 ± 15.3% and 71.7 ± 9.1% for homogenization and freeze-thawing, respectively). Such results illustrated the importance of using appropriate ISTDs to correct for signal losses or enhancement during sample preparation and data acquisition. Without ISTDs, the true metabolite concentration cannot be determined (Broadhurst et al. 2018).

As shown in Fig. 3, the PE value is a combination of sample preparation effect and matrix factor. Our results indicated that our sample preparation procedures yielded accurate and reproducible results for all metabolites, independently of the extraction process used. The matrix effects (not shown) showed similar trends as the PE values. Thus the loss or enhancement of some ISTDs and metabolites detected, based on PE values, were caused by the presence of the matrices.

For extraction of cell cultures, monolayers, or tissues, use of freeze-thawing cycle(s) or physical disruption (homogenization or ultrasonication) have been reported in literature without studies comparing these methods. Here, we compared freeze-thawing and homogenization extraction procedures to evaluate the most suitable sample preparation methods, based on recovery, efficiency, and repeatability. The two extraction procedures showed no significant differences in recovery, repeatability, and efficiency (Figs. 4, 5).

For sample preparation, extraction by homogenization required only 2 min, compared with approximatively 1 h required for extraction by freeze-thawing. However, the presence of beads used for homogenization required an additional transfer step to obtain clean extracts, separated from beads, protein, and cell debris. Without a drying–reconstitution step, both sample preparation techniques are simple and fast and comparable with “dilute and shoot” methods applied for the analysis of homogenous matrices (e.g., urine). Rapid, simple measures should decrease the risk of changes in the metabolome during sample preparation.

## Conclusions

These results demonstrated the importance of using ISTDs to correct for signal losses or enhancements caused by the presence of matrices. We showed that structurally related ISTDs can enable partial compensation for metabolite losses or enhancements, while isotopologue ISTDs enable full compensation for such artifacts. Additionally, the resulting ability to absolutely quantify analytes will improve inter-study and inter-laboratory comparisons.

Among the tobacco-related metabolites evaluated, the results for some were strongly affected by a drying–reconstitution step, some metabolites or ISTDs such as nicotine and nicotine D_3_ were not always detected after a drying step. For other compounds such as nornicotine, the drying step induced high RSD and low recoveries. By eliminating this step, favorable recoveries were obtained for those analytes. Furthermore, for all compounds assessed, there were no significant differences between homogenization- and freeze-thawing-based extractions.

The sample preparation methods evaluated here were rapid, easily performed, and gave repeatable results due to the ISTDs’ for complex adherent and suspended 3D cell cultures. The metabolite list is flexible, expendable and customizable according to study needs; thus the protocols and method presented herein can be useful for metabolomics investigation.
